# Unique roles of vaginal *Megasphaera* phylotypes in reproductive health

**DOI:** 10.1099/mgen.0.000526

**Published:** 2021-12-13

**Authors:** Abigail L. Glascock, Nicole R. Jimenez, Sam Boundy, Vishal N. Koparde, J. Paul Brooks, David J. Edwards, Jerome F. Strauss III, Kimberly K. Jefferson, Myrna G. Serrano, Gregory A. Buck, Jennifer M. Fettweis

**Affiliations:** ^1^​ Life Sciences, Virginia Commonwealth University, Richmond, VA, USA; ^2^​ Department of Microbiology & Immunology, Virginia Commonwealth University, Richmond, VA, USA; ^3^​ Center for Microbiome Engineering and Data Analysis, Virginia Commonwealth University, Richmond, VA, USA; ^4^​ Department of Supply Chain Management and Analytics, Virginia Commonwealth University, Richmond, VA, USA; ^5^​ Department of Statistical Sciences and Operations Research, Virginia Commonwealth University, Richmond, VA, USA; ^6^​ Department of Obstetrics and Gynecology, Virginia Commonwealth University, Richmond, VA, USA; ^7^​ Department of Computer Science, Virginia Commonwealth University, Richmond, VA, USA

**Keywords:** BV, Megasphaera, trichomoniasis, vaginal microbiome

## Abstract

The composition of the human vaginal microbiome has been extensively studied and is known to influence reproductive health. However, the functional roles of individual taxa and their contributions to negative health outcomes have yet to be well characterized. Here, we examine two vaginal bacterial taxa grouped within the genus *Megasphaera* that have been previously associated with bacterial vaginosis (BV) and pregnancy complications. Phylogenetic analyses support the classification of these taxa as two distinct species. These two phylotypes, *Megasphaera* phylotype 1 (MP1) and *Megasphaera* phylotype 2 (MP2), differ in genomic structure and metabolic potential, suggestive of differential roles within the vaginal environment. Further, these vaginal taxa show evidence of genome reduction and changes in DNA base composition, which may be common features of host dependence and/or adaptation to the vaginal environment. In a cohort of 3870 women, we observed that MP1 has a stronger positive association with bacterial vaginosis whereas MP2 was positively associated with trichomoniasis. MP1, in contrast to MP2 and other common BV-associated organisms, was not significantly excluded in pregnancy. In a cohort of 52 pregnant women, MP1 was both present and transcriptionally active in 75.4 % of vaginal samples. Conversely, MP2 was largely absent in the pregnant cohort. This study provides insight into the evolutionary history, genomic potential and predicted functional role of two clinically relevant vaginal microbial taxa.

## Data Summary

The genomes of *Megasphaera* phylotype 1 (MP1, strain M1-70), *Megasphaera* phylotype 2 (MP2, strain M2-4) and *Megasphaera* phylotype 2 (MP2, strain M2-8) were submitted to DDBJ/ENA/GenBank under accession numbers PTJT00000000, PTJU00000000 and PTJV00000000, respectively. The versions described in this paper are versions PTJT01000000, PTJU01000000 and PTJV01000000. Raw sequencing reads for *Megasphaera* phylotype 1 (MP1, strain M1-70), *Megasphaera* phylotype 2 (MP2, strain M2-4) and *Megasphaera* phylotype 2 (MP2, strain M2-8) have been submitted to SRA under accession numbers SRR15600837, SRR15600836 and SRR15600835, respectively. Data from the VaHMP has been deposited under dbGAP Study Accession phs000256.v3.p2. Raw metatranscriptomic sequences from the MOMS-PI project are available at NCBI’s controlled-access dbGaP (Study Accession: phs001523.v1.p1). Access to additional fields can be requested through the RAMS Registry (https://ramsregistry.vcu.edu). Custom code for the analysis is now available on GitHub at the following location: https://githubcom/Vaginal-Microbiome-Consortium./Megasphaera.

**Impact Statement**

Vaginal microbiome composition is linked with risk of preterm birth and HIV. Megasphaera phylotypes 1 and 2 (MP1, MP2) have been associated with bacterial vaginosis (BV) and related adverse reproductive health sequelae. However, these taxa had not previously been characterized beyond their 16S rRNA genes and associations. This study provides an in-depth characterization of these two phylotypes spanning genomic structure, predicted function, phylogenetic history, clinical and demographic associations and transcriptional profiling. These analyses suggest that MP1 and MP2 represent two distinct species that have evolved to become more host-associated and potentially adapted to the vaginal niche. The two species differed in their predicted metabolic capabilities, suggesting the potential for differential roles within the vaginal environment. MP1 was associated with BV whereas MP2 was associated with trichomoniasis suggesting differences in clinical associations. MP1, which previously has been linked to preterm birth, was maintained in pregnancy and more transcriptionally active than predicted based on its abundance. This study provides a framework for understanding the phylogenetic lineage and the functional roles of vaginal *Megasphaera *. It also highlights the importance of distinguishing between MP1 and MP2 in microbial surveys and further examining the role of MP1 in preterm birth.

## Introduction

The vaginal microbiome is an important determinant of women’s reproductive health, pregnancy outcomes and neonatal health [1–6]. Optimal vaginal microbial health is typically characterized by dominance of one or more lactic-acid producing species of the genus *Lactobacillus* that function to lower the pH and prohibit the growth of other organisms [7]. A vaginal microbiome depleted of protective vaginal lactobacilli and enriched in diverse anaerobic species is often clinically diagnosed as bacterial vaginosis (BV). BV is the most common vaginal condition worldwide, affecting an estimated 27 % of women in North America [8]. This condition has been associated with an increased risk of acquiring sexually transmitted infections (STIs) as well as pregnancy complications including spontaneous preterm birth [9–13]. While associations of vaginal microbial taxa with reproductive health conditions such as BV are well established, the pathophysiological significance of these taxa remains largely unknown. Developing a more comprehensive understanding of how individual taxa contribute to negative health outcomes is essential for understanding the underlying biological mechanisms and for the development of effective therapeutics.

Here we focus on two vaginal anaerobic taxa, *Megasphaera* phylotype 1 (MP1) and *Megasphaera* phylotype 2 (MP2), and their roles in reproductive health and disease. Both MP1 and MP2 have been previously associated with bacterial vaginosis across multiple cohorts [3, 14–16]. Due to its high specificity for the condition, MP1 has been used in combination with other taxa for molecular diagnosis of BV [15, 17]. MP2 was described by Martin *et al.* to be more prevalent in samples collected from women with trichomoniasis, suggesting the potential for divergent roles of these vaginal *Megasphaera* in disease states [18]. *Megasphaera* species have also been linked to an increased risk for HIV acquisition [19, 20]. Given that MP1 and MP2 have been observed in the urogenital tracts of adolescent males and heterosexual couples, it seems likely that these bacteria can be sexually transmitted [21, 22].

Vaginal carriage of *Megasphaera* is strongly associated with BV, and pregnant women with BV have an elevated risk for spontaneous preterm birth [23]. The outcomes across antibiotic intervention studies for prevention of preterm birth have been inconsistent, which may be attributed in part to the significant heterogeneity in study design and the choice and timing of therapeutic intervention [23]. It is now clear that there are different subtypes of BV that can be stratified using molecular approaches, and some subtypes of BV may be more tightly linked to preterm birth than others. Even though BV has long been linked to elevated risk for preterm birth, more recent vaginal microbiome studies have identified higher MP1 carriage in women who go on to deliver preterm [12, 24–26]. Interestingly, Mitchell *et al.* observed MP1 in samples collected from the upper genital tract of women undergoing hysterectomy [27], suggesting that MP1 may be capable of ascending from the vaginal environment into the upper genital tract. Together, these observations suggest that MP1 can colonize the vaginal environment, ascend into the upper genital tract and potentially contribute to PPROM and/or spontaneous preterm birth.

In the current study, we use several approaches to delineate the roles of MP1 and MP2 in reproductive health. These include phylogenetic analyses that probe the evolutionary history of these organisms, genomic characterization that permits assessment of their metabolic potential, and a study to define their individual associations with demographic and clinical measures.

## Methods

### Cultivation of MP1 and MP2

Using anaerobic technique, we cultivated, isolated and sequenced the genomes of one isolate of *Megasphaera* phylotype 1 (MP1, strain M1-70) and two isolates of *Megasphaera* phylotype 2 (MP2, strains M2-4 and M2-8) from frozen glycerol stocks of vaginal swab samples collected through the Vaginal Human Microbiome Project (VaHMP) [28]. One mid-vaginal swab from each participant was used to inoculate 1.0 ml of supplemented brain-heart infusion (sBHI) culture media with an added cryo-protectant (20 % glycerol) and stored at −80** **°C (Table S1, available in the online version of this article). Frozen vaginal culture samples were targeted for cultivation based on the presence and high relative abundance of bacterial targets of interest. These samples were identified using 16S rRNA gene-based vaginal microbiome profiles generated for each participant. A scraping of the frozen vaginal culture media from the selected targets was used to inoculate agar plates for bacterial culture. Scrapings were plated on both ThermoScientific Remel Chocolate agar (lysed blood agar) and ThermoScientific Remel Brucella Blood agar (5 % sheep’s blood) at four dilutions: 1 : 10, 1 : 100, 1 : 1000 and 1 : 10 000. Plates were stored at 37 °C for 24–48 h. The plates were enclosed in three nested Ziploc bags along with a Mitsubishi Anaeropack-Anaero packet to simulate anaerobic conditions. Individual colonies were selected for growth and purification from the dilution plates based on colony morphology and differential growth characteristics. After re-streaking for visibly pure colonies, the isolates were taxonomically identified by colony PCR amplification of the full 16S rRNA gene using universal 16S primers [29, 30]. Amplicons were purified using the Qiagen QIAquick PCR Purification Kit and sequenced using the Applied Biosystems 3730 DNA Analyzer. Colonies that were identified as bacterial targets of interest and exhibited no evidence of contamination were selected for extraction of genomic DNA. A single colony inoculum was added to 5 ml of sBHI in a 15 ml falcon tube. Tubes were loosely capped to allow gas exchange and stored in a rack at 37 °C for 24–48 h in three nested Ziploc bags containing a Mitsubishi Anaeropack-Anaero. The DNA was then extracted using the Qiagen DNeasy Blood & Tissue Kit and quantified using the Nanodrop 2000 spectrophotometer. Frozen stocks for MP1 and MP2 isolates were not recoverable.

### Genome sequencing and assembly

Purified genomic DNA from the single MP1 isolate was sequenced using the Roche 454 GS FLX Titanium platform. The resulting reads were trimmed for quality and assembled using Newbler v2.8 [31]. Purified genomic DNA derived from the two MP2 isolates M2-4 and M2-8 were sequenced using the Illumina MiSeq platform and the resulting reads were trimmed for quality and assembled using Newbler v2.8, CLCBio and SPAdes [31–33]. These three assemblies were merged using CISA to produce the most complete and accurate contigs [34].

### Structural genomic analysis

Genomic synteny was analysed between genome representatives of MP1 and MP2 and other host-associated *Megasphaera* species. This analysis was performed at the both the protein and nucleic acid level. Nucleic acid-based synteny analyses were performed using NUCmer while amino acid-based synteny analyses were performed using PROmer. Both NUCmer and PROmer are available as a part of the MUMmer 3.0 package [35]. Synteny plots were created using gnuplot from the gnuplot 4.2 package and MUMmerplot, which is also available as a part of the MUMmer 3.0 package [36]. Genomic GC composition was determined using in-house scripts. Codon usage within the genomes was calculated using cusp, a programme included in the EMBOSS Tools package available through EMBL-EBI [37]. Comparative analyses of basic genome statistics including genome size, predicted number of proteins and GC composition were performed using a Kruskal–Wallis test. This was performed using the kruskal.test function in R. All calculated *P* values were adjusted using the FDR correction in R using the p.adjust function. Resulting corrected *q* values are reported in Results.

### Measures of genomic similarity

Analyses were performed utilizing three MP1 genomes, three MP2 genomes and all publicly available *Megasphaera* and *Anaeroglobus* genomes at NCBI as of 1 January 2015 (Table S2). One metagenomic *Megasphaera elsdenii* assembly was excluded from the analysis due to variation in size and gene content from other deposited *M. elsdenii* genomes. To assess genomic similarity using the entire nucleotide content of the genomes, a pairwise calculation of the average nucleotide identity was performed using a publicly available script (https://github.com/chjp/ANI). The 16S ribosomal RNA gene sequences are commonly used to distinguish bacterial species and establish evolutionary relatedness [38]. The 16S rRNA gene sequences were identified and extracted from genomes using RNAmmer [39]. Sequence similarity of the 16S rRNA genes was determined using the blastn algorithm [40]. In order to delineate genus boundaries, pairwise percentage of conserved protein (POCP) values were calculated using in-house scripts developed based on the methods described in Qin *et al.* [41].

### CSI and CSP detection

Conserved signature proteins (CSPs) and genomic regions containing conserved signature indels (CSIs) were identified using blast [40]. Genomic regions containing CSIs were aligned using muscle and visualized using Jalview [42, 43]. This analysis was based on work performed by Campbell *et al.* identifying CSPs and CSIs indicative of the placement of certain taxa within the class Negativicutes [44].

### Genome annotation and metabolic reconstruction

Genomes were annotated using both an in-house annotation pipeline and rast [40], a web-based genome visualization, annotation and metabolic reconstruction tool provided by NMPDR [45]. As a part of the in-house genome annotation pipeline, the following programmes were used. Genes were called using both Glimmer3 and GeneMarkS [46, 47]. Ribosomal RNA genes were identified and extracted from genomes using RNAmmer [39]. Genes encoding tRNAs were identified in genomes using tRNAScan-SE [48]. Orthologous genes were detected using rpsblast in conjunction with Pfam and COG databases [40, 49–52]. Predicted gene functions were annotated using blastx and the nr database at NCBI [40]. Metabolic reconstruction was performed using ASGARD [50] and visual representations of predicted variation within metabolic pathways were generated using the programme color-maps [53]. To determine genes lost in MP1 and MP2, genes specific to MP1 and MP2 and genes that can be used to distinguish the two phylotypes, rast annotation was utilized. Findings were verified by comparing rast results to the genome annotation pipeline Glimmer3 and GeneMarkS gene calls. Further verification was performed using the tblastn algorithm to compare known annotated protein sequences available through NCBI to the raw genomic contigs [40, 49, 52]. OrthoDB, an online database for orthologous groups was used to identify orthologous genes [54].

### Phylogenetic analysis

To perform a phylogenetic reconstruction of the 16S rRNA gene, 16S rRNA sequences were identified and extracted from the genomes using RNAmmer [39]. The extracted 16S rRNA gene sequences were aligned using muscle [ 42]. The resulting alignment file was converted to phylip format using a web-based tool for DNA and protein file format conversion, ALignment Transformation EnviRonment or ALTER [55]. RAxML-HPC was used to perform a rapid bootstrap analysis using 1000 bootstraps and search for the best scoring maximum-likelihood tree using the gamma model of heterogeneity [56]. To create a phylogenetic reconstruction of all Negativicutes class genomes, 145 orthologous genes were used. OrthoDB, an online database for orthologous groups was used to determine which orthologous genes were conserved at the family level (Veillonellaceae) [54]. These genes were verified using reciprocal blast and extracted from the six MP1 and MP2 genomes as well as from all publicly available genomes classified to the class Negativicutes at NCBI as of 1 January 2015. *Clostridium botulinum* A strain Hall was selected as the outgroup. This species was chosen due to its classification in the same phylum (Firmicutes) but different class (Clostridia versus Negativicutes) as compared to the Negativicutes genomes. Each orthologous gene was separately aligned using muscle, a programme within the EMBOSS Tools package available through EMBL-EBI [37, 42]. Alignments were visually examined and those with large gaps or likely errors were discarded. Sequences from all orthologs were concatenated together to form one large informative sequence. Concatenated sequences were then pruned for informative regions using Gblocks [57]. The resulting sequences were converted from pir to phylip format using the web tool, ALTER [55]. RAxML-HPC was used to perform a rapid bootstrap analysis using 100 bootstraps and search for the best scoring maximum-likelihood tree using optimization of substitution rates, the gamma model of heterogeneity and the WAG amino acid substitution matrix [56]. Aesthetic changes to the tree were made using TreeDyn [58].

### Sample collection, vaginal microbiome profiling and analysis

Samples collected as part of the Vaginal Human Microbiome Project (VaHMP) at Virginia Commonwealth University were used for this study as previously described [28]. Briefly, mid-vaginal wall swab samples were collected and DNA was extracted from the swabs using the MoBio Powersoil DNA Isolation Kit. DNA samples were randomized to avoid batch effects and the V1-V3 region of the 16S rRNA gene was amplified using PCR and universal primers (Table S3) [29, 30]. The amplified 16S rDNA fragments were sequenced using the Roche 454 GS FLX Titanium platform. Sequences were classified using both the Ribosomal Database Project (RDP) classifier and the in-house STIRRUPS (Species-level Taxon Identification of rDNA Reads using a USEARCH Pipeline Strategy) classifier to achieve species-level classification (version 10-18-17) [59, 60]. Samples that yielded less that 5000 reads were excluded from analysis.

Taxa were determined to be present if they comprised at least 0.1 % of the vaginal microbiome profile of a given sample. Demographics and health history data was self-reported by the participants. Associations were calculated based on the presence or absence of a taxon of interest (threshold of 0.1 % of total reads) in combination with given demographic or clinical data. Statistical significance was calculated using a generalized linear model using logistic regression as implemented in the ‘glm’ function in R. All calculated *P* values were corrected for multiple testing using the FDR correction method. This was performed in R using the p.adjust function. Adjusted *q* values are reported in Results.

### Alpha diversity measures

16S rDNA-based vaginal microbiome profiles from the Vaginal Human Microbiome Project (VaHMP) outpatient cohort of non-pregnant subjects was used for this analysis (*n*=3091). Alpha diversity for each microbiome profile was calculated using relative proportion data, renormalized to exclude unclassified reads (below 97 % threshold). Inverse Simpson’s index was used as the measure of alpha diversity. This metric was calculated using the R package ‘vegan’. Average inverse Simpson’s index alpha diversity measures were generated for four subsets of vaginal microbiome data: (i) samples containing neither phylotype, (ii) samples containing only MP1, (iii) samples containing only MP2 and (iv) samples containing both MP1 and MP2. Presence of a phylotype was denoted by a relative abundance of greater than or equal to 0.1 % of the vaginal microbiome profile. Statistical significance was calculated using a two-tailed Student’s *t*-test with a significance level of 0.05.

### Relative risk

The non-pregnant, outpatient VaHMP cohort was used for this analysis (*n*=3091). Samples met the threshold of at least 5000 reads. Vaginal infection status was determined based on clinician diagnosis at time of visit. Relative risk values and their corresponding 95 % confidence interval values were calculated based on the standard relative risk formula. Relative risk = (*A*/*A*+*B*) / (*C*/*C*+*D*) where *A* represents the number of samples where the taxon is present and the participant is diagnosed with the disease, *B* represents the number of samples where the taxon is present but the participant is not diagnosed with the disease, *C* represents the number of samples where the taxon is absent but the participant is diagnosed with the disease and *D* represents the number of samples where the taxon is not present and the participant is not diagnosed with the disease.

### Pregnancy analysis

A case-matched cohort was used for this analysis. A cohort of 779 pregnant women was case-matched 1 : 1 based on ethnicity, age and income to 779 non-pregnant controls. Using the R package ‘wilcox’, we performed a Mann–Whitney U test on all vaginal microbial taxa present in at least 5 % of samples that comprise at least 0.1 % relative proportion of the microbiome profile. We utilized the R function ‘p.adjust’ to correct for multiple testing using the FDR correction. Results for three *Lactobacillus* species, MP1, MP2 and select associated organisms associated with dysbiosis are shown [61].

### Transcriptomic analyses

The Multi-Omic Microbiome Study-Pregnancy Initiative (MOMS-PI) Preterm Birth cohort was utilized for this analysis [62]. This cohort consists of several hundred thousand samples collected from pregnant women throughout and after their pregnancies. For meta-transcriptomics, we collected a mid-vaginal swab from each participant and pre-processed the sample within an hour of collection by inserting the swab into RNAlater (Qiagen). These swabs were then processed using MoBio Power Microbiome RNA Isolation kit as described by the manufacturer. Total RNA was depleted of human and microbial rRNA using the Epicentre/Illumina Ribo-Zero Magnetic Epidemiology Kit as described by the manufacturer. Enriched messenger RNA was prepared for sequencing by constructing cDNA libraries using the KAPA Biosystems KAPA RNA HyperPrep Kit. Indexed cDNA libraries were pooled in equimolar amounts and sequenced on the Illumina HiSeq 4000 instrument running four multiplexed samples per lane with an average yield of ~100 Gb/lane, sufficient to provide >100× coverage of the expression profiles of the most abundant 15–20 taxa in a sample. Raw sequence data was demultiplexed into sample-specific fastq files using *bcl2fastq* conversion software from Illumina. Adapter residues were trimmed from both 5′ and 3′ end of the reads using Adapter Removal tool v2.1.3. The sequences were trimmed for quality using *meeptools* [63], retaining reads with minimum read length of 70b and *meep* (maximum expected error) quality score less than 1. Human reads were identified and removed from each sample by aligning the reads to hg19 build of the human genome using the BWA aligner [64]. Transcripts were classified using HUMAnN2 [65, 66] and shortBRED [67]. Transcripts assigned to either MP1 or MP2 were analysed for this study.

## Results

### Evolutionary history and genomic divergence of MP1 and MP2

**Fig. 1.**
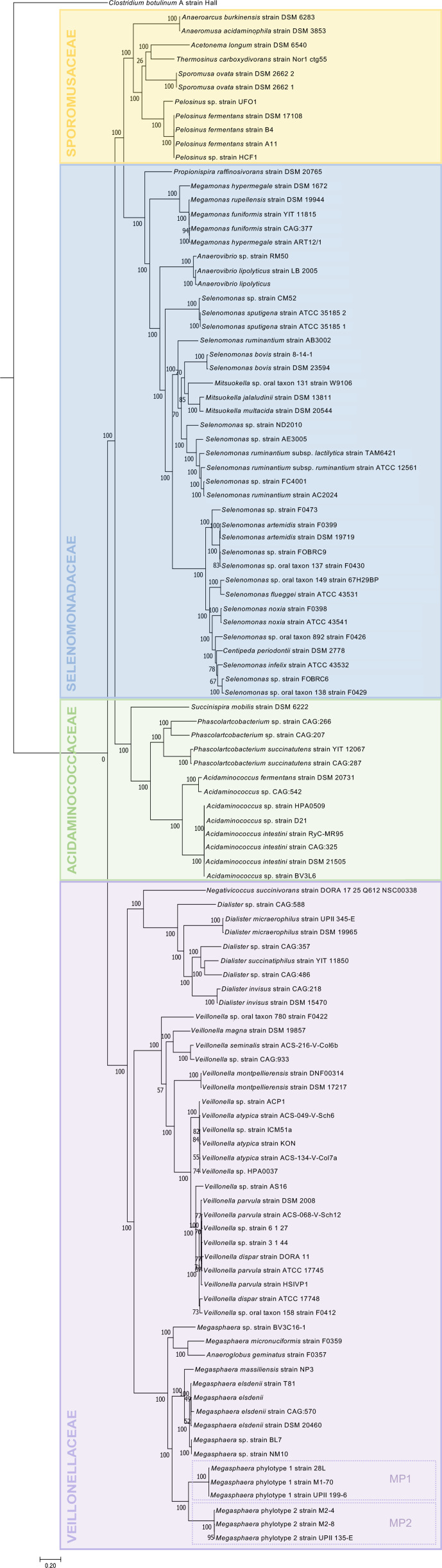
Maximum-likelihood phylogenetic tree of the class negativicutes. A total of 145 orthologous genes from 110 genomes assigned to the class Negativicutes were included in this analysis. *Clostridium botulinum* A strain Hall was designated as the outgroup. This maximum-likelihood phylogenetic tree was generated using 100 bootstrap replicates. Bootstrap values as present at nodes of the tree. Families within the tree highlighted in different colours: Sporomusaceae: yellow, Selenomonadaceae: blue, Acidaminococcaceae: green, Veillonellaceae: purple. MP1 and MP2 genomes are outlined with dotted lines and labelled.

**Fig. 2.**
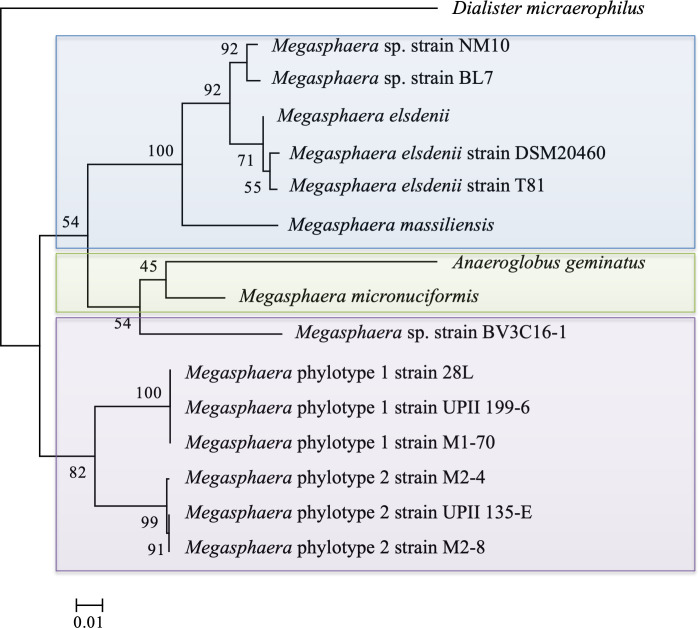
Maximum-likelihood phylogenetic tree of 16S ribosomal RNA gene. A maximum-likelihood tree with 1000 bootstraps was generated from full-length 16S ribosomal RNA gene sequences (nucleotide). Numbers at nodes are indicative of bootstrap support of that node placement. *Dialister micraerophilus* was selected as the outgroup and is a human oral isolate also classified in the family Veillonellaceae. Remaining isolates are coloured by their site of isolation: blue – mammalian gut, green – human oral, purple – human vaginal.

Genomes of three representative MP1 isolates and three representative MP2 isolates were analysed to gain insight into the mechanisms underlying their colonization of the human vaginal environment (Table S4). A phylogenetic analysis of 145 orthologous genes using 110 genomes classified to the class Negativicutes revealed that MP1 and MP2 are evolutionarily distinct and separated from the nearest *Megasphaera */* Anaeroglobus* clade (Fig. 1). Similar results were observed in a phylogenetic analysis using 16S ribosomal RNA (rRNA) genes and the inferred topology was largely reflective of niche adaptation (Fig. 2). In one case, niche-specific separation did not occur; *Megasphaera* sp. BV3C16-1, which was isolated from the human vagina, was grouped with oral taxa. This taxon has been reported in vaginal microbiome [25], but it has been observed at low abundance and prevalence. For example, the taxon was identified in five of 3870 vaginal samples (0.12 %) at a threshold of 0.01 % in a cohort of women enrolled through the Vaginal Human Microbiome Project (VaHMP) [68].

Given the significant divergence of these two vaginal phylotypes from other closely related taxa, we performed a percentage of conserved protein (POCP) analysis, a metric for delineating genus boundaries [41]. The suggested cutoff for delineation of genera is a POCP value of less than 50 % to the genus type strain. The POCP values for members of the MP1 and MP2 clade in comparison to the type strain (* Megasphaera elsdenii* DSM 20460) range from 49.6–52.6 % (Fig. S1, Table S5). *Anaeroglobus geminatus,* currently classified as a separate genus, had a POCP value of 52.5 % compared to the *Megasphaera* type strain [69]. A recent study by Campbell *et al.* identified CSIs and CSPs used to classify organisms to families within the class Negativicutes [44]. We identified all CSIs and CSPs indicative of Veillonellaceae family genomes in MP1 and MP2 genomes (Table S6), supporting their previous placement within the Veillonellaceae family. However, three of nine CSP markers specific for the class Negativicutes were absent from all MP1 and MP2 genomes, indicative of genome reduction that is not observed in other host-related *Megasphaera *. While biochemical analyses have yet to be performed, the phylogeny, POCP analysis, loss of CSP markers, and specificity of the clade to the vaginal environment could support placement of these phylotypes into a novel genus of bacteria.

**Fig. 3.**
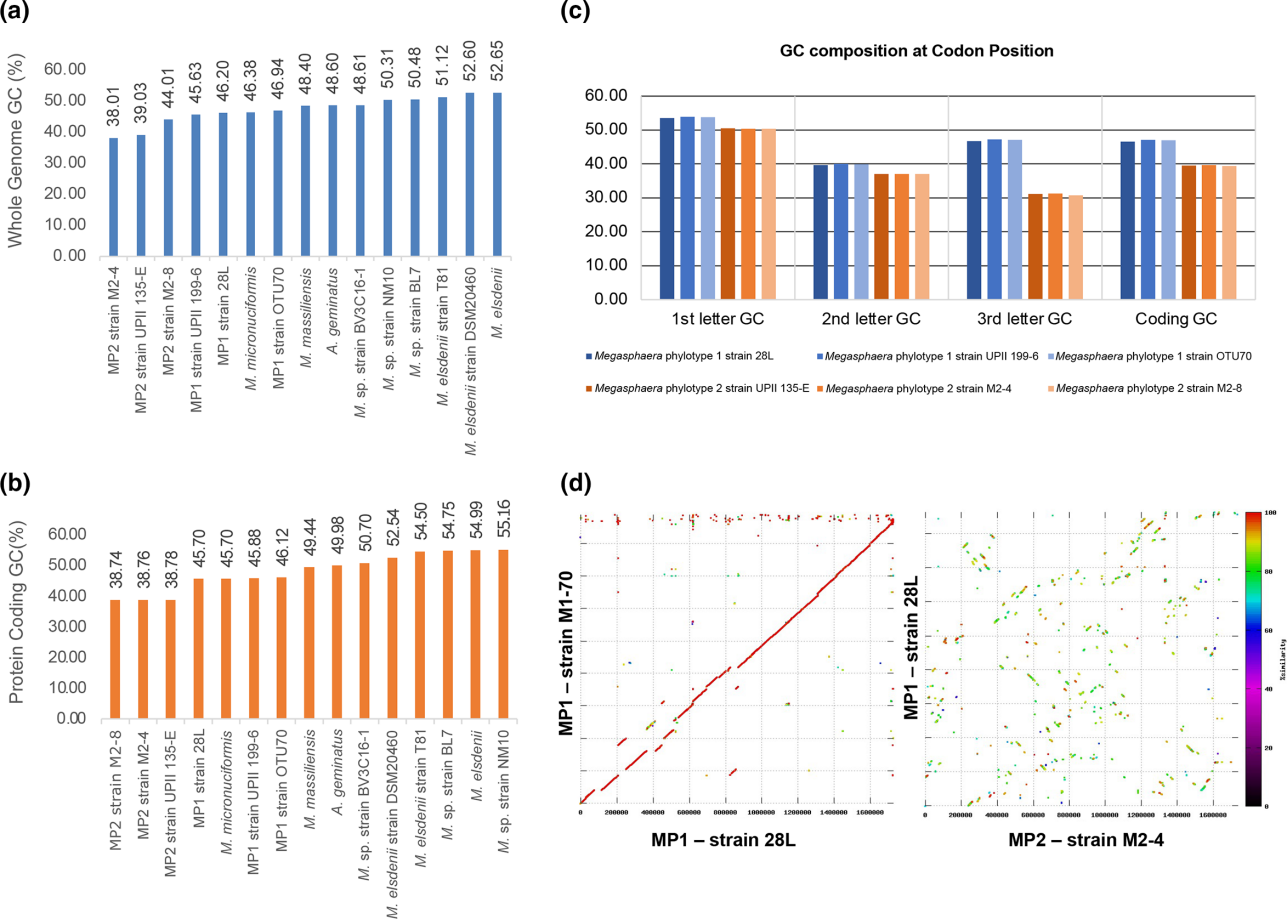
Distinctive GC composition, codon preference and genomic structure between vaginal *Megasphaera* phylotypes. Differences in both (a) whole genome and (b) protein-coding GC composition are shown. (c) Codon preference is distinct between MP1 and MP2 genomes based on differences in GC composition at specific codon positions and in the overall coding GC composition (MP1: blue, MP2: orange). (d) Synteny is conserved within phylotype but lost between MP1 and MP2 genomes. Synteny plots demonstrate structural alignment of genomic content at the amino acid level. Colour designates similarity at the amino acid level. The left panel shows the strong conservation of genomic synteny and protein identity (red colour) between two MP1 genomes. The right panel show massive genome rearrangement and loss of amino acid sequence conservation between a MP1 and a MP2 genome.

We compared the genomes of MP1 and MP2 with genomes of seven *Megasphaera* isolates from human and mammalian GI tracts, the single human oral *Anaeroglobus* isolate and the vaginal *Megasphaera* sp. BV3C16-1 isolate [69–73]. All of the MP1 and MP2 isolates exhibit evidence of genome reduction with an average genome size of 1.71 megabases (Mb) relative to an average genome size of 2.35 Mb for the other studied *Megasphaera* and *Anaeroglobus* genomes (*q*=0.001, 95 % CI [−0.97 Mb, −0.32 Mb], Kruskal–Wallis test for differences in genome size with FDR correction). The MP1 and MP2 genomes contain a predicted 1571 protein-coding genes on average, which is significantly fewer than the number of protein-coding genes for the other studied *Megasphaera* and *Anaeroglobus* genomes, which contained an average of 2116 genes (*q*=0.00015, 95 % CI [−749 genes, −341 genes], Kruskal–Wallis test for differences in predicted gene count with FDR correction). MP1 and MP2 also exhibit lower average GC composition with an average of 42.6 % compared to an average of 51.1 % in the other host-associated genomes in the *Megasphaera/Anaeroglobus* clade (*q*=0.0005, 95 % CI [−12.17%, −4.75%], Kruskal–Wallis test for differences in average GC composition with FDR correction) (Fig. 3a). Reduction in genome size and lower GC percentage has been observed in vaginal strains of other bacterial taxa including *Lactobacillus* and *Gardnerella *, suggesting reductive evolution may be a common feature of adaptation to the vaginal environment [74, 75].

One representative of MP1 (Veillonellaceae bacterium DNF00751) and two representatives of MP2 (* Megasphaera* genomosp. 2, Veillonellaceae bacterium KA00182) were deposited after our analyses were complete. ANI values suggest that they are similar in genomic content to the genome representatives analysed in this study. Veillonellaceae bacterium DNF00751 had ANI values ranging from 96.5–98.6 % compared to the three MP1 genomes utilized in our analysis. *Megasphaera* genomosp. 2 had ANI values ranging from 98.5–99.0 % and Veillonellaceae bacterium KA00182 had ANI values ranging from 98.6–99.0 % to the three MP2 genomes utilized in our analyses.

### Taxonomic placement of MP1 and MP2 as two discrete species

Similarity of the 16S rRNA gene at an identity threshold of 97 % is often used to delineate species. The 16S rRNA similarity between the two phylotypes is 96.3 %. This figure along with reports by Srinivasan *et al.*, implies that the two phylotypes are best classified as distinct species based on 16S rRNA gene sequence similarity (Table S7) [76, 77]. The average nucleotide identity (ANI) between MP1 and MP2, which takes into account the entire nucleotide content of genomes, is 73 %. This figure is markedly less than the 95–96 % threshold suggested for species demarcation using this method (Table S8) [78]. Our phylogenetic analyses (Figs 1 and 2) reflected these findings, with MP1 and MP2 identified as sister taxa, distinct from other *Megasphaera* and *Anaeroglobus* and separated by significant branch lengths, signifying extensive divergence.

Further comparative analyses revealed that genomic synteny is conserved within phylotype, with variations attributable to the presence of temperate bacteriophage. However, extensive genome rearrangement was observed between MP1 and MP2 genomes (Fig. 3c and S2). While a significant difference in genome size was not observed between MP1 (average of 1.72 Mb) and MP2 (average of 1.70 Mb) isolates (*q*=0.7497, 95 % CI [−0.118 Mb, 0.152 Mb], Kruskal–Wallis test for differences in genome size with FDR correction), there was an observed difference in GC composition between the MP1 (average of 46.3 %) and MP2 isolates (average of 39.0 %) (*q*=0.000002, 95 % CI [6.95 %, 7.61 %], Kruskal–Wallis test for differences in GC composition with FDR correction). The two phylotypes also exhibit GC-divergent codon preference at the third position (average GC composition at third position: MP1- 47 %, MP2- 31 %), signalling evolutionary pressure for a reduction in GC composition in MP2 (Fig. 3b). The observed sequence divergence, differential GC composition and codon preference, and lack of synteny between MP1 and MP2 genomes provide support for the designation of the two phylotypes as distinct species.

### Genomic evidence for niche specialization to the vaginal environment

To assess differences in the predicted metabolic potential, we annotated and performed metabolic reconstructions of 15 genomes including representatives of MP1, MP2 and related bacterial strains classified to the *Megasphaera* and *Anaeroglobus* genera. As expected, given the observed genome reduction of MP1 and MP2, many metabolic pathways present among all other related taxa are absent in the MP1/MP2 clade (Table S9). MP1 and MP2 are predicted to lack genes conserved in other *Megasphaera* and *Anaeroglobus* genomes that function to transport putrescine and spermidine, metabolize nitrogen, produce selenocysteine and transport and modify the metals nickel and molybdenum. Thus, these organisms may have evolved to rely on synergy with the host and/or microbial co-inhabitants. Interestingly, MP1 and MP2 are predicted to have retained the ability to produce spermidine, a known metabolic marker of BV [79]. Despite the overall genomic reduction of MP1 and MP2, these vaginal phylotypes have also gained functions specific to their clade. MP1 and MP2 specifically encode virulence genes including variable tetracycline resistance genes (i.e. *tetM*, *tetO*, *tetW*) and genes necessary for iron uptake (i.e. *tonB* and hemin uptake outer membrane receptor). Iron sequestration is commonly a critical characteristic of pathogenic bacteria and may be pertinent to the vaginal microbiome given the influx of available iron during menses [79, 80]. MP1 and MP2 genomes also encode multiple CRISPR-associated proteins, which likely function to protect these bacteria from foreign genetic elements [81].

### Predicted functional divergence of MP1 and MP2

MP1 and MP2 also possess unique predicted metabolic functions, indicative of their divergence. While genomes of both phylotypes encode the majority of genes required for glycolysis, MP2 genomes lack hexokinase. The absence of this gene suggests that MP2 strains cannot use glucose as a carbon source. MP1 genomes are predicted to lack adenosine deaminase (ADA), an enzyme involved in the adenine salvage pathway. In contrast, MP2 genomes retain ADA but lack the gene encoding cytidine deaminase, which functions in the recycling of cytosine bases. These differential salvage strategies are intriguing given that MP1 genomes have markedly higher GC content than MP2 genomes. The phylotypes also differ in their ability to synthesize amino acids. MP2 genomes are incapable of synthesizing leucine and tryptophan, while MP1 genomes lack the ability to interconvert serine and cysteine. Production of aromatic amino acids including tryptophan is energetically expensive [82]. Thus, the loss of tryptophan synthesis genes in MP2 is an example of energetically favourable genome reduction in this host-associated organism. Further biochemical assays with MP1 and MP2 isolates are necessary to confirm our genomic findings. Unfortunately, our repeated attempts to recover the isolates from frozen stocks were not successful, and we were thus unable to perform biochemical assays. Since our initial submission, Srinivasan *et al.* confirmed that none of the vaginal Megasphaera, including MP2, were able to use glucose as a carbon source [83].

### MP1 and MP2 phylotypes have distinct clinical associations

Given the distinct metabolic capacities of the MP1 and MP2 phylotypes, we examined the self-survey and clinical data associated with the Vaginal Human Microbiome Project (VaHMP) to investigate their individual roles in reproductive health [68]. We first examined demographic and clinical associations with vaginal MP1 and MP2 carriage in a cohort of 3091 non-pregnant women. Using a cutoff of 0.1 % relative abundance, 27 % of women in this cohort (845/3091) carried MP1 only, 5 % (163/3091) carried MP2 only, 6 % (182/3091) carried both phylotypes, and 62 % (1901/3091) carried neither phylotype. The cutoff of 0.1 % relative abundance was chosen to balance the detection of rare organisms with the potential for contamination and classification errors. If we define an organism as present in a microbiome profile based on one read, 35.3 % of women (1129/3190) carried MP1 only, representing an increase of 8.0 % (*n*=284). In total, 174/3190 (5.5 %) samples contained MP2 only, representing an increase of 0.2 % (*n*=11). Overall, 599/3091 (19.4 %) samples contained both MP1 and MP2, representing an increase of 13.5 % (*n*=417). Lastly, 1189/3091 (38.5 %) of samples contained zero reads attributable to MP1 or MP2, representing a decrease of 23 % (*n*=712). More sensitive methods would be necessary to definitively determine bacterial presence at this abundance. Thus, all following analyses are performed using the more conservative 0.1 % relative abundance cutoff to determine microbial presence.

**Fig. 4.**
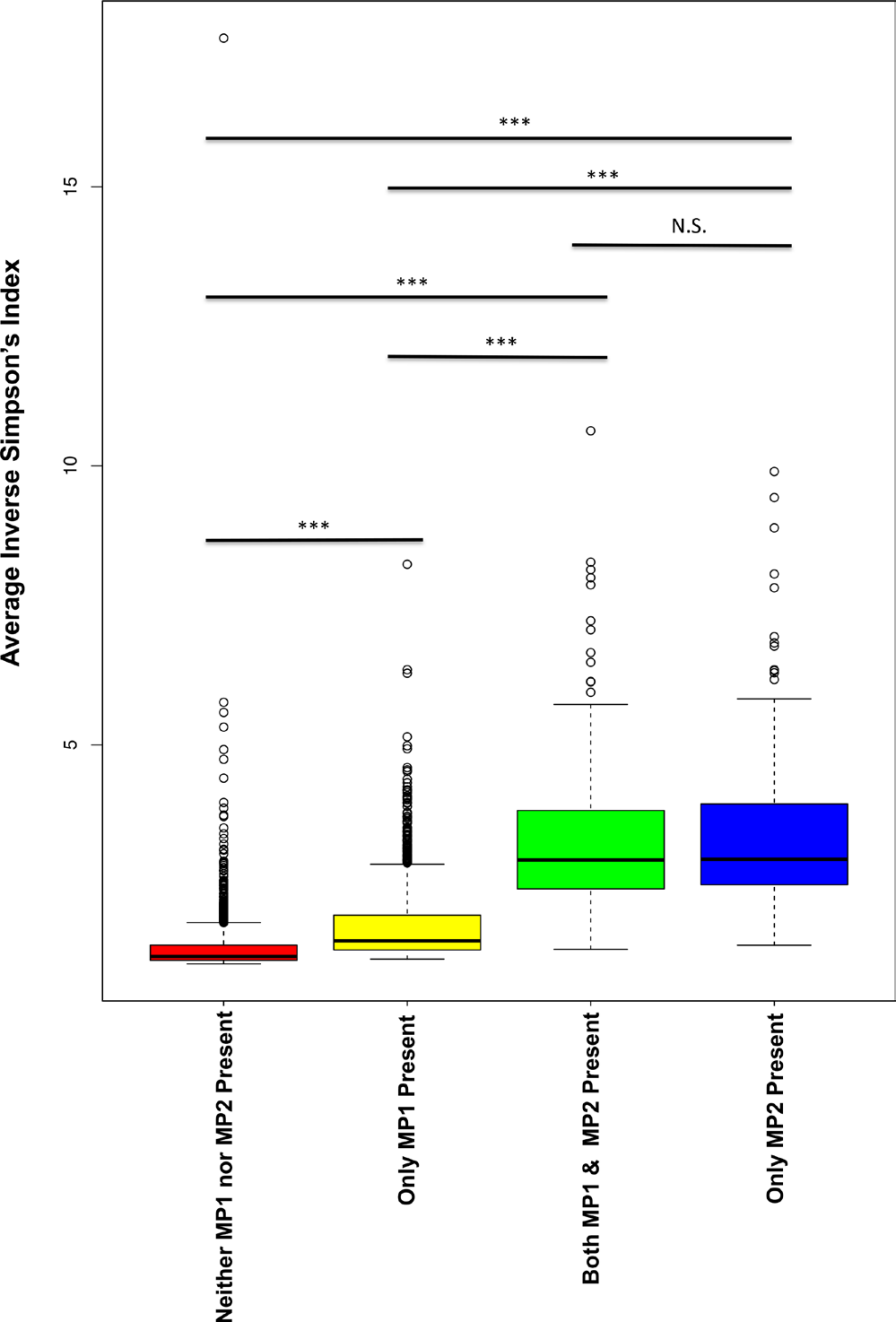
Vaginal *Megasphaera* phylotypes associated with increased alpha diversity. Alpha diversity of vaginal microbiome profiles was calculated using the inverse Simpson’s index. Distribution of inverse Simpson’s index for each group is shown. Boxes show median and interquartile ranges, with whiskers denoting maximum and minimum values. Outliers are shown as dots. Significance was determined using a two-tailed Student’s *t*-test. Four different groups are shown, samples containing neither MP1 or MP2 (*n*=1901, red), samples containing MP1 only (*n*=845, yellow), samples containing both MP1 and MP2 (*n*=182, green) and samples containing only MP2 (*n*=163, blue). Taxa were determined to be present in a sample if they comprised greater than or equal to 0.1 % of the sample. Samples with MP1 only, MP2 only and both phylotypes all exhibit increased alpha diveristy, with MP2 only samples being the most highly diverse. All comparisons were found to be highly signifcant (*P*<0.01) with the exception of MP2 only and both phylotypes.

Compared to the average alpha diversity (i.e. inverse Simpson’s index) of samples containing neither of the two phylotypes (1.37), alpha diversity was increased in samples containing MP1 only (1.79), MP2 only (3.47) and both phylotypes (3.37) (Fig. 4). Notably, vaginal microbiome communities containing MP2 exhibited an almost two-fold increase in alpha diversity compared with MP1 alone. Associations with demographics were determined using a generalized linear model. Both phylotypes were associated with African-ancestry (MP1: q=3.00e-31, MP2: q=1.10e-21, with FDR correction). The association of MP1 and MP2 with African ancestry is consistent with the increased incidence of BV and the previously reported higher rates of BV-type vaginal microbial communities among women with African ancestry [84–86]. MP1 and MP2 were significantly associated with a self-reported annual household income of less than 20 k (MP1: q=2.23e-18, MP2: *q*=3.31e-18 with FDR correction) (Table S10). Within this cohort, ethnicity and age were not independent of income. However, the associations between MP1 and MP2 and income were still significant among groups stratified by race (African ancestry vs. non-African ancestry). When age is analysed as a covariate, the associations between MP1 and MP2 and income are no longer statistically significant.

Fethers *et. al* previously reported that MP1 was associated with women who have sex with women (WSW) [3]. WSW experience higher rates of BV than women who do not have sex with women [87]. Thus, we examined the association of both *Megasphaera* phylotypes with WSW. Although 44 % (38/86) of women who reported a current female partner were MP1 positive and there was a positive association between WSW and MP1 carriage (coefficient=0.42, *z*=1.88, *q*=0.08 with FDR correction), it did not reach the threshold for significance (*P*<0.05). Using the generalized linear model (GLM), the race/ethnicity field was identified as a significant covariate with WSW. In stratified analyses, we found that among women who did not report African ancestry, there was a strong association between WSW (*N*=25) and MP1 (coefficient=1.36, *z*=3.33, *q*=0.001 with FDR correction), but that among women reporting African ancestry, WSW (*N*=61) was not significantly associated with MP1 (coefficient=−0.05, *z*=−0.19, *q*=0.85 with FDR correction). The majority of participants not reporting African ancestry self-identified as Caucasian (68 %). This finding highlights the need for precision medicine approaches that account for the contribution of individual environmental and genetic factors and their interactions to fully understand the contributions that shape vaginal microbiome composition and impact risk for adverse reproductive health outcomes.

To assess the association of these two phylotypes with three common vaginal infections (i.e. bacterial vaginosis, candidiasis and trichomoniasis) we performed a relative risk analysis. We observed that while both MP1 and MP2 were associated with an increased risk for BV (MP1 : 4.57, 95 % CI [3.76,5.55], MP2 : 2.19, 95 % CI [1.79–2.69]), MP1 is associated with a higher risk for this condition (Table 1). In contrast, MP2 was associated with an increased risk for trichomoniasis (4.84, 95 % CI [3.06–7.64]), whereas MP1 had no association (0.96, 95 % CI [0.59–1.56]). Using the GLM approach, MP1 and MP2 strains were both associated with self-reported vaginal odour (MP1: *q*=5.39e-18, MP2: *q*=1.36e-10 with FDR correction) and vaginal discharge (MP1: *q*=1.40e-17, MP2: *q*=4.64e-7 with FDR correction). Both phylotypes were also associated with clinician-diagnosed elevated vaginal pH (>4.5) (MP1: *q*=3.56e-34, MP2: *q*=7.29e-12 with FDR correction) consistent with previous reports. Carriage of MP1 and MP2 were also associated with having more than ten lifetime sexual partners (MP1: *q*=0.00037, MP2: *q*=4.65e-5 with FDR correction) and having more than one sexual partner in the past month (MP1: *q*=0.0002, MP2: *q*=2.24e-5 with FDR correction). These observed associations are consistent with the association of these phylotypes with BV.

**Table 1.** Relative risk of vaginal infections in the presence of MP1 and MP2 bacterial vaginosis trichomoniasis candidiasis

	Bacterial Vaginosis	Trichomoniasis	Candidiasis
*** Megasphaera* phylotype 1**	**4.57 (3.76–5.55)**	**0.96 (0.59–1.56)**	**0.52 (0.37–0.74)**
***Megasphaera*** **phylotype 2**	**2.19 (1.79–2.69)**	**4.84 (3.06–7.64)**	**0.54 (0.30–0.96)**
* Gardnerella vaginalis *	6.44 (4.18–9.92)	2.47 (1.23–4.94)	0.65 (0.49–0.88)
* Prevotella* cluster2	5.48 (4.31–6.98)	2.32 (1.44–3.74)	0.45 (0.33–0.62)
Clostridiales BVAB2	4.14 (3.46–4.96)	1.04 (0.63–1.72)	0.42 (0.28–0.64)
* Sneathia amnii *	3.97 (3.25–4.84)	2.93 (1.83–4.70)	0.48 (0.35–0.68)
* Mycoplasma hominis *	2.57 (2.15–3.08)	5.80 (3.70–9.09)	1.07 (0.75–1.53)
*‘Ca.* Mycoplasma girerdii’	0.88 (0.50–1.55)	21.00 (13.82–31.91)	0.71 (0.27–1.87)

Relative risk values were calculated based on the customary relative risk formula for MP1, MP2 and taxa known to be associated with BV and trichomoniasis. The relative risk conferred by each taxon is shown with the 95 % confidence interval in parentheses for BV, trichomoniasis and yeast infection (Candidiasis). Our total cohort of non-pregnant women (*N*=3091) was used for this analysis.

### MP1 and MP2 in pregnancy

**Fig. 5.**
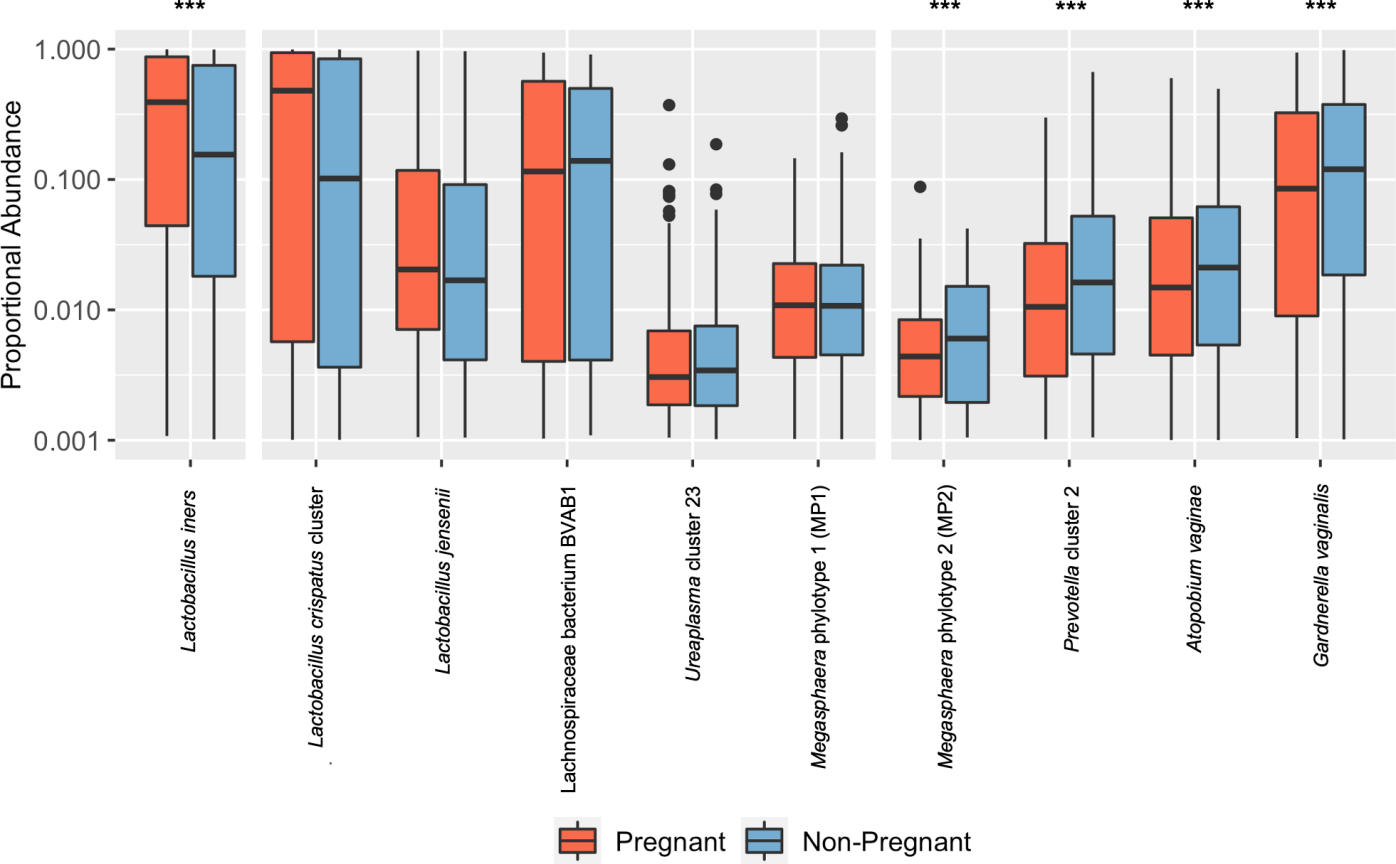
*
Megasphaera* phylotype 1 (MP1) not significantly excluded in pregnancy. Results were generated from a cohort of 779 pregnant women case matched 1 : 1 with non-pregnant controls (*N*=1558). The distribution of the proportional abundance of taxa of interest is shown in both pregnant (red) and non-pregnant (blue) cohorts. Boxes show median and interquartile ranges, with whiskers denoting maximum and minimum values. Outliers are shown as dots. Significance was determined using a Mann–Whitney U test with FDR correction. *Lactobacillus iners* is shown to be significantly more prevalent in the pregnant cohort (*q*=1.20E-6). *Lactobacillus crispatus* cluster*, Lactobacillus jensenii,* Lachnospiraceae BVAB1, *Megasphaera* phylotype 1 (MP1) and *Ureaplasma* cluster 23 are not significantly different between the two cohorts (*q*=0.19, 0.23, 0.43, 0.56, 0.26, respectively). *Megasphaera* phylotype 2 (MP2), *Prevotella* cluster 2, *Atopobium vaginae* and *Gardnerella vaginalis* are significantly lower in the pregnant cohort (*q*=5.82×10^−3^, 6.09E-8, 7.95E-8, 2.82E-7, respectively).

Recent studies have shown that the vaginal microbiome in pregnancy is associated with decreased alpha diversity and dominance of protective *Lactobacillus* species [88–91]. Similarly, BV-associated organisms have been shown to be less prevalent in pregnant women [68, 92]. Thus, not surprisingly in a case-matched cohort of 779 pregnant and 779 non-pregnant women from the VaHMP study, we found that MP2 was significantly decreased in pregnancy (*q*=0.00585, Mann–Whitney U test with FDR correction) (Fig. 5). This finding is in agreement with previous work demonstrating that BV organisms are often less prevalent in pregnancy [68, 92]. In contrast, MP1 was not significantly excluded in the pregnant cohort (*q*=0.596, Mann–Whitney U test with FDR correction). MP1 has been previously associated with risk for preterm birth [12, 24, 25]; additional studies will be necessary to determine whether the ability of MP1 to persist throughout gestation has implications for complications in pregnancy.

To determine whether the two vaginal phylotypes were functionally active in pregnancy, we analysed metatranscriptomic data from 57 samples collected from 52 pregnant women who delivered at term as a part of the case-control preterm birth cohort from the Multi-‘Omic Microbiome Study – Pregnancy Initiative (MOMS-PI) [24]. This is a reanalysis of a subset of an existing dataset previously published in 2019 [24, 92]. In this cohort, 43 samples contained transcripts assigned to MP1 while only one sample contained transcripts assigned to MP2 (Tables S11 and S12), consistent with our observation that MP2 seems to be less prevalent in pregnancy while MP1 is maintained. Because MP2 was only detected in a single sample, we will focus on the findings pertaining to MP1 here. The data showed that *in vivo* in the vaginal environment, MP1 strains transcribed genes from 34 unique pathways (Table S11). Notably, MP1 strains transcribed genes involved in butyrate production, which has previously been associated with BV [79].

**Fig. 6.**
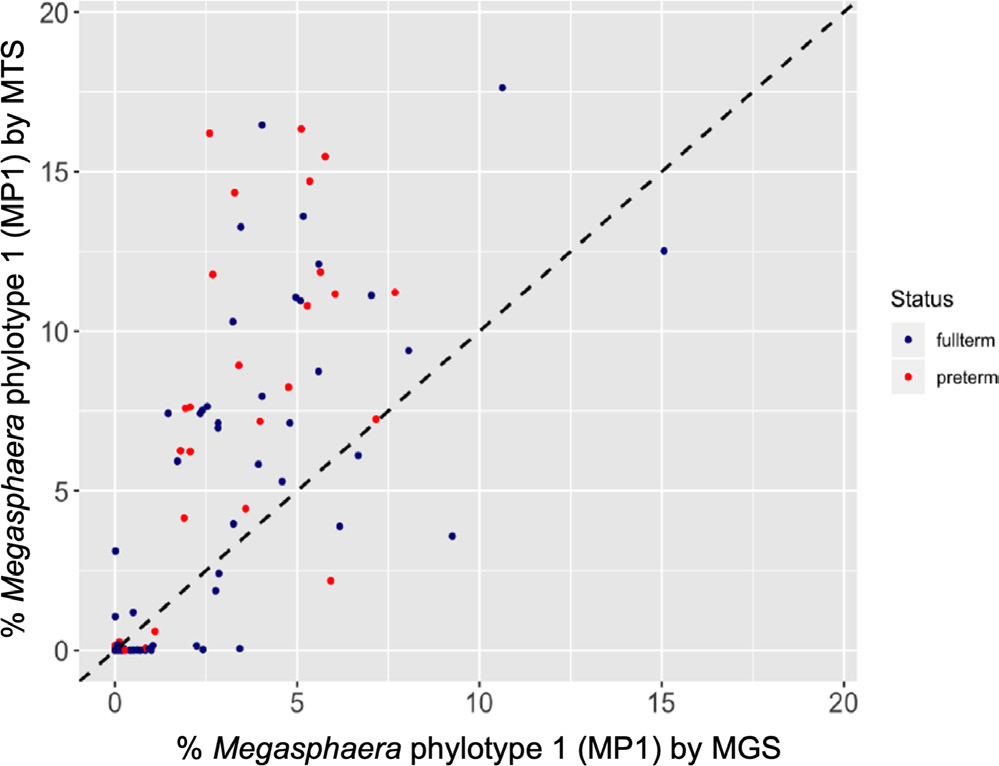
Relationship between *Megasphaera* 16S read abundance and transcript abundance in paired datasets. Results were generated from samples collected from a cohort of pregnant women that participated in the MOMS-PI study. Samples were processed for whole metagenome microbiomics and transcriptomics. Percent of total transcripts attributed to the taxon of interest is shown on the *y*-axis. Percent of total whole metagenome sequencing reads attributed to the taxon of interest are shown on the *x*-axis. Each dot represents an individual sample. The relationship between WMGS and WMTS representation of *Megasphaera* phylotype 1 (MP1) is shown. Data points representing samples from women who went on to deliver full term are shaded blue, while data points representing samples from women who went on to deliver preterm are shaded red. The dotted line extending across the graph diagonally represent the expected 1 : 1 relationship of WMGS and WMTS- based abundance measures.

For this cohort (*N*=57), we also had paired 16S rDNA profiles and metagenome sequencing profiles generated as a part of a previous study [24]. In these paired data, we observed that the 16S rDNA relative abundance measures for MP1 were strongly correlated to their paired metagenomic relative abundance measures (*ρ*=0.92, Spearman’s rank correlation). This finding supports the use of 16S rDNA profiles in lieu of metagenomic sequencing data to estimate the relative abundance of MP1 in these cohorts. The correlation of MP1 metagenomic relative abundance measures to their paired metatranscriptomic relative abundance measures was also significant (*ρ*=0.91, Spearman’s rank correlation). Intriguingly, the relative abundance measures of the transcripts assigned to MP1 were greater than the observed relative abundance measures in the paired metagenomic dataset (*P*=2.95e-05, Mann–Whitney U test) (Fig. 6). This suggests that MP1 is highly transcriptionally active in these samples and makes up a greater proportion of the transcripts than would be predicted based upon the metagenomic data alone. Taken together, the above analyses demonstrate that MP1 is maintained in pregnancy, in contrast to other BV-associated organisms, and is transcriptionally active in a majority of pregnant women in our cohort. These observations in combination with previous associations of MP1 with PPROM and spontaneous preterm labour, highlights MP1 as an important target for future study [12, 24, 25].

## Discussion

Our phylogenetic analyses suggest that MP1 and MP2 are evolutionarily divergent from other *Megasphaera* species as well as each other. We provide strong evidence that supports placement of MP1 and MP2 into separate genus. Definitive confirmation of this would require comprehensive biological and physiological studies. Compared to other *Megasphaera *, both organisms exhibit loss of gut-specific metabolic pathways, acquisition of iron uptake pathways, and loss of genes involved in the biosynthesis of differential amino acids. These organisms also exhibit reduced genomes and lowered GC composition, indicative of a transition to a more host-dependent state [85] that seems to be a common feature of adaption to the vaginal environment [73, 74]. Taken together these observations are suggestive of adaptation to the host and/or vaginal environment.

Several lines of evidence support the hypothesis that MP1 and MP2 have adapted from an ancestral host-associated gastrointestinal or oral strain to colonize the vaginal niche: (i) the similarity of MP1 and MP2 to human gastrointestinal and oral *Megasphaera* species, (ii) the ubiquity of *Megasphaera* in the gastrointestinal and oral sites of humans and mammals [44, 70], (iii) the streamlined genomes of MP1 and MP2, a common feature of strains identified in the human vagina, and (iv) the ease of physical transfer of microorganisms from the mouth and/or rectum to the vagina. Based on our observations, we hypothesize that these two phylotypes share a host-associated common ancestor, likely a colonizer of a gastrointestinal or oral site. Their evolutionary divergence is characterized by progressive gene loss and genome reduction, common features among host-dependent organisms. These changes may be indicative of host dependence and/or adaptation to the vaginal environment specifically.

MP1 and MP2 are evolutionarily divergent and functionally distinct from one another as well, and these findings have important implications for the contributions of these unique phylotypes to vaginal infections and pregnancy complications [3, 12–14, 17, 24]. Several lines of evidence show differential associations of these two phylotypes with clinical diagnoses and demographic factors. As expected, our analyses confirmed that MP1 is tightly correlated with BV as diagnosed by Amsel’s criteria in a cohort of 3091 non-pregnant women of reproductive age. This result is consistent with numerous previous studies that have demonstrated the strong association of MP1 with BV and led to its use as a biomarker for the diagnosis of the condition. MP2 was also associated with BV [RR=2.19, 95 % CI (1.79–2.69)] in the cohort, but to a lesser extent than MP1 [RR=4.57, 95 % CI (3.76–5.55)]. This finding is consistent with previous reports of the specificity and sensitivity of MP1 and MP2 for BV diagnosis. While MP1 and MP2 have both been reported to have high specificity for BV ranging from 88.5–98.1 % for MP1 and 98.9–100 % for MP2 as diagnosed by Amsel’s criteria, Nugent score or a combination of both diagnostic measures, the sensitivity of MP2 (6.9 –31.0 %) has been reported to be significantly lower than that of MP1 (68.4–95.1 %) [17, 93]. In the current study, we observed an overall prevalence of 33.2 % (*n*=1027) for MP1 and 11.2 % (*n*=345) for MP2 among non-pregnant women of reproductive age. However, our results also suggest that the two major *Megasphaera* phylotypes may be associated with different subtypes of vaginal dysbiosis.

In the cohort of 3091 non-pregnant women of reproductive age, MP2 was strongly associated with trichomoniasis whereas MP1 was not associated with the condition. To our knowledge, the association of MP2 with trichomoniasis was first described by Martin *et al.* in 2013, and our study confirms and extends this observation [18]. Martin *et al.* also highlighted an observation from a 1992 study suggesting that *Trichomonas vaginalis* infection was associated with intermediate flora as defined by Nugent score among pregnant women [94]. Together, these findings highlight the need to distinguish between related taxa, such as MP1 and MP2, in microbiome analyses in order to accurately define the functionally relevant subtypes of vaginal dysbiosis and how they contribute to adverse reproductive health outcomes.

In the current study, MP1 and MP2 were both more prevalent among women who reported African ancestry (MP1 AA: 41.1 %, MP1 Non-AA: 19.8 %, MP2 AA: 15.9 %, MP2 Non-AA: 3.1 %), consistent with several previous reports including an analysis of the first 1686 women enrolled in the VaHMP cohort [1, 68, 84]. The association of MP1 and MP2 with African ancestry is consistent with the increased incidence of BV among women with African ancestry [84–86]. In a recent study of 33 white women and 16 black women who were BV negative as assayed by both Amsel’s and Nugent’s criteria, Beamer *et al.* did not detect significant differences in the colonization and density of a number of bacterial species assayed by cultivation and molecular methods [95]. Notably, organisms such as MP1 are rarely observed in women with low Nugent scores; MP1 was identified in 2/33 (6.1 %) white women and 2/16 black women (12.5 %) by qPCR. Nugent score, which calculates a score for BV based on the presence of bacterial morphotypes as assayed by microscopy, is a direct measure of microbial composition. By excluding individuals with higher Nugent score, a significant proportion of women of African ancestry may be excluded from the study. In this current analysis of the VaHMP cohort, race and ethnicity were tightly correlated with a number of covariates including measures of socioeconomic status such as education and annual income. Additional studies will be needed to further define the contributions of both genetic and environmental factors that shape vaginal microbiome composition [86, 96, 97].

We also found both MP1 and MP2 to be associated with increased alpha diversity of the microbiome profile and elevated vaginal pH (>4.5), which is one of Amsel’s criteria for BV and is consistent with previous studies linking these *Megasphaera* phylotypes to BV. Interestingly, the vaginal microbiome profile of women who carried MP2 exhibited higher alpha diversity compared to the vaginal microbiome of women who carried MP1 alone. MP2 exhibits greater genome reduction than MP1, likely making it more reliant on other microbial species and/or host factors. This genomic reduction may account for why MP2 is less prevalent in the overall population and specific to a more diverse dysbiotic state.

MP1 is prevalent in the vaginal environment and has been associated with preterm birth in several recent studies, marking it as a taxon of interest [23–25, 87]. Our current study suggests that MP1 levels are similar among pregnant and non-pregnant women, unlike many other BV-associated vaginal taxa, which seem to be excluded during the gestational shaping of the vaginal microbiome [26, 98, 99]. MP1 is highly prevalent in the VaHMP cohort, colonizing 33.2 % of women in the study. These findings suggest that this highly prevalent organism colonizes the vaginal environment and remains present and transcriptionally active during pregnancy. Mitchell *et al.* observed MP1 in the upper genital tract (UGT) of women undergoing hysterectomy suggesting that this organism is likely capable of ascending into the UGT. This capability combined with the ability of MP1 to maintain colonization during pregnancy suggests that this organism is a candidate for future studies investigating the proposed model where ascending infection of vaginal organisms contributes to in preterm labour and/or birth. *Megasphaera* has also been associated with low vitamin D levels [100] highlighting a possible link between vaginal microbiome signatures and host state [101]. Identifying mechanisms that permit this organism to pervade the changing vaginal environment associated with the progression of pregnancy may possibly lead to the development of more effective preventative therapeutics targeting microbe-related preterm labour and delivery. Our study also highlights the need for continued exploration of mechanisms of microbial evolution in the human microbiome. Understanding the processes that underlie adaptation to specific human host-associated environments will inform strategies for modulating the microbiome to prevent disease and promote human microbial health.

## Supplementary Data

Supplementary material 1

Supplementary material 2

## References

[1] Ravel J, Gajer P, Abdo Z, Schneider GM, Koenig SSK (2011). Vaginal microbiome of reproductive-age women. Proc Natl Acad Sci U S A.

[2] Romero R, Gómez R, Chaiworapongsa T, Conoscenti G, Kim JC (2001). The role of infection in preterm labour and delivery. Paediatr Perinat Epidemiol.

[3] Fethers K, Twin J, Fairley CK, Fowkes FJI, Garland SM (2012). Bacterial vaginosis (BV) candidate bacteria: associations with BV and behavioural practices in sexually-experienced and inexperienced women. PloS One.

[4] van de Wijgert JHHM (2017). The vaginal microbiome and sexually transmitted infections are interlinked: Consequences for treatment and prevention. PLoS Med.

[5] Dominguez-Bello MG, Costello EK, Contreras M, Magris M, Hidalgo G (2010). Delivery mode shapes the acquisition and structure of the initial microbiota across multiple body habitats in newborns. Proc Natl Acad Sci U S A.

[6] van Best N, Hornef MW, Savelkoul PHM, Penders J (2015). On the origin of species: Factors shaping the establishment of infant’s gut microbiota. Birth Defect Res C.

[7] Tachedjian G, Aldunate M, Bradshaw CS, Cone RA (2017). The role of lactic acid production by probiotic *Lactobacillus* species in vaginal health. Res Microbiol.

[8] Peebles K, Velloza J, Balkus JE, McClelland RS, Barnabas RV (2019). High global burden and costs of bacterial vaginosis: a systematic review and meta-analysis. Sex Transm Dis.

[9] Cohen CR, Lingappa JR, Baeten JM, Ngayo MO, Spiegel CA (2012). Bacterial Vaginosis Associated with Increased Risk of Female-to-Male HIV-1 transmission: a prospective cohort analysis among African couples. PLoS Med.

[10] Hillier SL, Krohn MA, Cassen E, Easterling TR, Rabe LK (1995). The role of bacterial vaginosis and vaginal bacteria in amniotic fluid infection in women in preterm labor with intact fetal membranes. Clinical Infectious Diseases.

[11] Kenyon C, Colebunders R, Crucitti T (2013). The global epidemiology of bacterial vaginosis: a systematic review. Am J Obstet Gynecol.

[12] Nelson DB, Hanlon A, Nachamkin I, Haggerty C, Mastrogiannis DS (2014). Early pregnancy changes in bacterial vaginosis-associated bacteria and preterm delivery. Paediatr Perinat Epidemiol.

[13] Ravel J, Moreno I, Simón C (2021). Bacterial vaginosis and its association with infertility, endometritis, and pelvic inflammatory disease. Am J Obstet Gynecol.

[14] Zozaya-Hinchliffe M, Martin DH, Ferris MJ (2008). Prevalence and abundance of uncultivated Megasphaera-like bacteria in the human vaginal environment. Appl Environ Microbiol.

[15] Datcu R, Gesink D, Mulvad G, Montgomery-Andersen R, Rink E (2014). Bacterial vaginosis diagnosed by analysis of first-void-urine specimens. J Clin Microbiol.

[16] Lennard K, Dabee S, Barnabas SL, Havyarimana E, Blakney A (2018). Microbial composition predicts genital tract inflammation and persistent bacterial vaginosis in South African adolescent females. Infect Immun.

[17] Fredricks DN, Fiedler TL, Thomas KK, Oakley BB, Marrazzo JM (2007). Targeted PCR for detection of vaginal bacteria associated with bacterial vaginosis. J Clin Microbiol.

[18] Martin DH, Zozaya M, Lillis RA, Myers L, Nsuami MJ (2013). Unique vaginal microbiota that includes an unknown Mycoplasma-like organism is associated with Trichomonas vaginalis infection. J Infect Dis.

[19] McClelland RS, Lingappa JR, Srinivasan S, Kinuthia J, John-Stewart GC (2018). Evaluation of the association between the concentrations of key vaginal bacteria and the increased risk of HIV acquisition in African women from five cohorts: a nested case-control study. Lancet Infect Dis.

[20] Sabo MC, Lehman DA, Wang B, Richardson BA, Srinivasan S (2020). Associations between vaginal bacteria implicated in HIV acquisition risk and proinflammatory cytokines and chemokines. Sex Transm Infect.

[21] Zozaya M, Ferris MJ, Siren JD, Lillis R, Myers L (2016). Bacterial communities in penile skin, male urethra, and vaginas of heterosexual couples with and without bacterial vaginosis. Microbiome.

[22] Nelson DE, Dong Q, Van Der Pol B, Toh E, Fan B (2012). Bacterial communities of the coronal sulcus and distal urethra of adolescent males. PloS One.

[23] Lamont RF (2015). Advances in the prevention of infection-related preterm birth. Front Immunol.

[24] Fettweis JM, Serrano MG, Brooks JP, Edwards DJ, Girerd PH (2019). The vaginal microbiome and preterm birth. Nat Med.

[25] Paramel Jayaprakash T, Wagner EC, van Schalkwyk J, Albert AYK, Hill JE (2016). High Diversity and Variability in the Vaginal Microbiome in Women following Preterm Premature Rupture of Membranes (PPROM): A Prospective Cohort Study. PloS One.

[26] Hočevar K, Maver A, Vidmar Šimic M, Hodžić A, Haslberger A (2019). Vaginal microbiome signature is associated with spontaneous preterm delivery. Front Med.

[27] Mitchell CM, Haick A, Nkwopara E, Garcia R, Rendi M (2015). Colonization of the upper genital tract by vaginal bacterial species in nonpregnant women. Am J Obstet Gynecol.

[28] Fettweis JM, Serrano MG, Girerd PH, Jefferson KK, Buck GA (2012). A new era of the vaginal microbiome: advances using next-generation sequencing. Chem Biodivers.

[29] Frank JA, Reich CI, Sharma S, Weisbaum JS, Wilson BA (2008). Critical evaluation of two primers commonly used for amplification of bacterial 16S rRNA genes. Appl Environ Microbiol.

[30] Romero R, Hassan SS, Gajer P, Tarca AL, Fadrosh DW (2014). The vaginal microbiota of pregnant women who subsequently have spontaneous preterm labor and delivery and those with a normal delivery at term. Microbiome.

[31] Miller JR, Koren S, Sutton G (2010). Assembly algorithms for next-generation sequencing data. Genomics.

[32] Krämer A, Green J, Pollard J, Tugendreich S (2014). Causal analysis approaches in Ingenuity Pathway Analysis. Bioinformatics.

[33] Bankevich A, Nurk S, Antipov D, Gurevich AA, Dvorkin M (2012). SPAdes: A New Genome Assembly Algorithm and Its Applications to Single-Cell Sequencing. J Comput Biol.

[34] Lin S-H, Liao Y-C, Watson M (2013). CISA: Contig Integrator for Sequence Assembly of Bacterial Genomes. PLoS ONE.

[35] Delcher AL, Phillippy A, Carlton J, Salzberg SL (2002). Fast algorithms for large-scale genome alignment and comparison. Nucleic Acids Res.

[36] gnuplot homepage accessed 2015 http://gnuplot.info/.

[37] Rice P, Longden I, Bleasby A (2000). EMBOSS: the European Molecular Biology Open Software Suite. Trends Genet.

[38] Janda JM, Abbott SL (2007). 16S rRNA Gene Sequencing for Bacterial Identification in the Diagnostic Laboratory: Pluses, Perils, and Pitfalls. J Clin Microbiol.

[39] Lagesen K, Hallin P, Rødland EA, Staerfeldt H-H, Rognes T (2007). RNAmmer: consistent and rapid annotation of ribosomal RNA genes. Nucleic Acids Res.

[40] Altschul SF, Gish W, Miller W, Myers EW, Lipman DJ (1990). Basic local alignment search tool. J Mol Biol.

[41] Qin Q-L, Xie B-B, Zhang X-Y, Chen X-L, Zhou B-C (2014). A proposed genus boundary for the prokaryotes based on genomic insights. J Bacteriol.

[42] Edgar RC (2004). MUSCLE: multiple sequence alignment with high accuracy and high throughput. Nucleic Acids Res.

[43] Waterhouse AM, Procter JB, Martin DMA, Clamp M, Barton GJ (2009). Jalview Version 2--a multiple sequence alignment editor and analysis workbench. Bioinformatics.

[44] Campbell C, Adeolu M, Gupta RS (2015). Genome-based taxonomic framework for the class Negativicutes: division of the class Negativicutes into the orders Selenomonadales emend., Acidaminococcales ord. nov. and Veillonellales ord. nov. International Journal of Systematic and Evolutionary Microbiology.

[45] Overbeek R, Olson R, Pusch GD, Olsen GJ, Davis JJ (2014). The SEED and the Rapid Annotation of microbial genomes using Subsystems Technology (RAST). Nucleic Acids Res.

[46] Delcher AL, Harmon D, Kasif S, White O, Salzberg SL (1999). Improved microbial gene identification with GLIMMER. Nucleic Acids Res.

[47] Besemer J, Lomsadze A, Borodovsky M (2001). GeneMarkS: a self-training method for prediction of gene starts in microbial genomes. Implications for finding sequence motifs in regulatory regions. Nucleic Acids Res.

[48] Lowe TM, Eddy SR (1997). tRNAscan-SE: a program for improved detection of transfer RNA genes in genomic sequence. Nucleic Acids Res.

[49] Edgar RC (2010). Search and clustering orders of magnitude faster than BLAST. Bioinformatics.

[50] Finn RD, Mistry J, Tate J, Coggill P, Heger A (2010). The Pfam protein families database. Nucleic Acids Res.

[51] Tatusov RL (2003). The COG database: an updated version includes eukaryotes. BMC Bioinformatics.

[52] Marchler-Bauer A, Panchenko AR, Shoemaker BA, Thiessen PA, Geer LY (2002). CDD: a database of conserved domain alignments with links to domain three-dimensional structure. Nucleic Acids Res.

[53] Alves JMP, Buck GA (2007). Automated System for Gene Annotation and Metabolic Pathway Reconstruction Using General Sequence Databases. Chem Biodivers.

[54] Kriventseva EV, Tegenfeldt F, Petty TJ, Waterhouse RM, Simão FA (2015). OrthoDB v8: update of the hierarchical catalog of orthologs and the underlying free software. Nucleic Acids Res.

[55] Glez-Peña D, Gómez-Blanco D, Reboiro-Jato M, Fdez-Riverola F, Posada D (2010). ALTER: program-oriented conversion of DNA and protein alignments. Nucleic Acids Res.

[56] Stamatakis A (2014). RAxML version 8: a tool for phylogenetic analysis and post-analysis of large phylogenies. Bioinformatics.

[57] Castresana J (2000). Selection of conserved blocks from multiple alignments for their use in phylogenetic analysis. Mol Biol Evol.

[58] Chevenet F, Brun C, Bañuls A-L, Jacq B, Christen R (2006). TreeDyn: towards dynamic graphics and annotations for analyses of trees. BMC Bioinformatics.

[59] Fettweis JM, Serrano MG, Sheth NU, Mayer CM, Glascock AL (2012). Species-level classification of the vaginal microbiome. BMC Genomics.

[60] Cole JR, Wang Q, Cardenas E, Fish J, Chai B (2009). The Ribosomal Database Project: improved alignments and new tools for rRNA analysis. Nucleic Acids Res.

[61] Whitney J (1997). Notes on methodology Testing for differences with the nonparametric mann-whitney U test. Journal of WOCN.

[62] Integrative HMP (iHMP) Research Network Consortium (2014). The Integrative Human Microbiome Project: dynamic analysis of microbiome-host omics profiles during periods of human health and disease. Cell Host Microbe.

[63] Koparde VN, Parikh HI, Bradley SP, Sheth NU (2017). MEEPTOOLS: a maximum expected error based FASTQ read filtering and trimming toolkit. IJCBDD.

[64] Li H, Durbin R (2010). Fast and accurate long-read alignment with Burrows–Wheeler transform. Bioinformatics.

[65] Ordoukhanian P, Nichols J, Head SR (2018). Primer Extension, Capture, and On-Bead cDNA Ligation: An Efficient RNAseq Library Prep Method for Determining Reverse Transcription Termination Sites. Methods Mol Biol Clifton NJ.

[66] Abubucker S, Segata N, Goll J, Schubert AM, Izard J (2012). Metabolic reconstruction for metagenomic data and its application to the human microbiome. PLoS Comput Biol.

[67] Kaminski J, Gibson MK, Franzosa EA, Segata N, Dantas G (2015). High-Specificity Targeted Functional Profiling in Microbial Communities with ShortBRED. PLoS Comput Biol.

[68] Fettweis JM, Brooks JP, Serrano MG, Sheth NU, Girerd PH (2014). Differences in vaginal microbiome in African American women versus women of European ancestry. Microbiology (Reading).

[69] Carlier J-P, Marchandin H, Jumas-Bilak E, Lorin V, Henry C (2002). Anaeroglobus geminatus gen. nov., sp. nov., a novel member of the family Veillonellaceae. Int J Syst Evol Microbiol.

[70] Rogosa M (1971). Transfer of Peptostreptococcus elsdenii Gutierrez et al. to a New Genus, Megasphaera [M. elsdenii (Gutierrez et al.) comb. nov.]. International Journal of Systematic Bacteriology.

[71] Padmanabhan R, Lagier J-C, Dangui NPM, Michelle C, Couderc C (2013). Non-contiguous finished genome sequence and description of Megasphaera massiliensis sp. nov. Stand Genomic Sci.

[72] Marchandin H, Jumas-Bilak E, Gay B, Teyssier C, Jean-Pierre H (2003). Phylogenetic analysis of some Sporomusa sub-branch members isolated from human clinical specimens: description of Megasphaera micronuciformis sp. nov. Int J Syst Evol Microbiol.

[73] Shetty SA, Marathe NP, Lanjekar V, Ranade D, Shouche YS (2013). Comparative genome analysis of Megasphaera sp. reveals niche specialization and its potential role in the human gut. PloS One.

[74] Mendes-Soares H, Suzuki H, Hickey RJ, Forney LJ (2014). Comparative functional genomics of Lactobacillus spp. reveals possible mechanisms for specialization of vaginal lactobacilli to their environment. J Bacteriol.

[75] Yeoman CJ, Yildirim S, Thomas SM, Durkin AS, Torralba M (2010). Comparative Genomics of Gardnerella vaginalis Strains Reveals Substantial Differences in Metabolic and Virulence Potential. PLoS One.

[76] Mende DR, Sunagawa S, Zeller G, Bork P (2013). Accurate and universal delineation of prokaryotic species. Nat Methods.

[77] Srinivasan S, Beamer MA, Fiedler TL, Austin MN, Sizova MV (2018). Characterization of novel megasphaera species from the female reproductive tract. American Journal of Obstetrics and Gynecology.

[78] Kim M, Oh H-S, Park S-C, Chun J (2014). Towards a taxonomic coherence between average nucleotide identity and 16S rRNA gene sequence similarity for species demarcation of prokaryotes. Int J Syst Evol Microbiol.

[79] Srinivasan S, Morgan MT, Fiedler TL, Djukovic D, Hoffman NG (2015). Metabolic signatures of bacterial vaginosis. mBio.

[80] Cornelissen CN, Hollander A (2011). TonB-Dependent Transporters Expressed by Neisseria gonorrhoeae. Front Microbiol.

[81] Koonin EV, Makarova KS (2017). Mobile Genetic Elements and Evolution of CRISPR-Cas Systems: All the Way There and Back. Genome Biol Evol.

[82] Priya VK, Sarkar S, Sinha S (2014). Evolution of tryptophan biosynthetic pathway in microbial genomes: a comparative genetic study. Syst Synth Biol.

[83] Srinivasan S, Beamer MA, Fiedler TL, Austin MN, Sizova MV (2019). Megasphaera lornae sp. nov., Megasphaera hutchinsoni sp. nov., and Megasphaera vaginalis sp. nov.: novel bacteria isolated from the female genital tract. Int J Syst Evol Microbiol.

[84] Marrazzo JM (2011). Interpreting the epidemiology and natural history of bacterial vaginosis: are we still confused?. Anaerobe.

[85] Ness RB, Hillier S, Richter HE, Soper DE, Stamm C (2003). Can known risk factors explain racial differences in the occurrence of bacterial vaginosis?. J Natl Med Assoc.

[86] Borgdorff H, van der Veer C, van Houdt R, Alberts CJ, de Vries HJ (2017). The association between ethnicity and vaginal microbiota composition in Amsterdam, the Netherlands. PloS One.

[87] Plummer EL, Vodstrcil LA, Fairley CK, Tabrizi SN, Garland SM (2019). Sexual practices have a significant impact on the vaginal microbiota of women who have sex with women. Sci Rep.

[88] MacIntyre DA, Chandiramani M, Lee YS, Kindinger L, Smith A (2015). The vaginal microbiome during pregnancy and the postpartum period in a European population. Sci Rep.

[89] Romero R, Hassan SS, Gajer P, Tarca AL, Fadrosh DW (2014). The composition and stability of the vaginal microbiota of normal pregnant women is different from that of non-pregnant women. Microbiome.

[90] Aagaard K, Riehle K, Ma J, Segata N, Mistretta T-A (2012). A metagenomic approach to characterization of the vaginal microbiome signature in pregnancy. PloS One.

[91] Walther-António MRS, Jeraldo P, Berg Miller ME, Yeoman CJ, Nelson KE (2014). Pregnancy’s Stronghold on the Vaginal Microbiome. PloS One.

[92] Serrano MG, Parikh HI, Brooks JP, Edwards DJ, Arodz TJ (2019). Racioethnic diversity in the dynamics of the vaginal microbiome during pregnancy. Nat Med.

[93] Hilbert DW, Smith WL, Chadwick SG, Toner G, Mordechai E (2016). Development and Validation of a Highly Accurate Quantitative Real-Time PCR Assay for Diagnosis of Bacterial Vaginosis. J Clin Microbiol.

[94] Hillier SL, Krohn MA, Nugent RP, Gibbs RS (1992). Characteristics of three vaginal flora patterns assessed Gram stain among pregnant women. American Journal of Obstetrics and Gynecology.

[95] Beamer MA, Austin MN, Avolia HA, Meyn LA, Bunge KE (2017). Bacterial species colonizing the vagina of healthy women are not associated with race. Anaerobe.

[96] Taylor BD, Totten PA, Astete SG, Ferris MJ, Martin DH (2018). Toll-like receptor variants and cervical Atopobium vaginae infection in women with pelvic inflammatory disease. Am J Reprod Immunol.

[97] Murphy K, Mitchell CM (2016). The Interplay of Host Immunity, Environment and the Risk of Bacterial Vaginosis and Associated Reproductive Health Outcomes. J Infect Dis.

[98] Chu DM, Seferovic M, Pace RM, Aagaard KM (2018). The microbiome in preterm birth. Best Pract Res Clin Obstet Gynaecol.

[99] Brown RG, Al-Memar M, Marchesi JR, Lee YS, Smith A (2019). Establishment of vaginal microbiota composition in early pregnancy and its association with subsequent preterm prelabor rupture of the fetal membranes. Translational Research.

[100] Jefferson KK, Parikh HI, Garcia EM, Edwards DJ, Serrano MG (2019). Relationship between vitamin D status and the vaginal microbiome during pregnancy. J Perinatol.

[101] (2019). Integrative HMP (iHMP) Research Network Consortium. The Integrative Human Microbiome Project Nature.

