# Supplementary material for: Unique roles of vaginal Megasphaera phylotypes in reproductive health
Source: Microb Genom. 2021 Dec 13;7(12):000526. doi: 10.1099/mgen.0.000526 (PMC8767330; doi:10.1099/mgen.0.000526)
Supplement: Supplementary material 1 [file mgen-7-0526-s001.pdf]

Supplementary Table 1. Supplemented Brain-Heart Infusion Recipe

Ingredient	Quantity
Brain-Heart Infusion Powder (Oxoid)	9.25g
Yeast Extract	2.50g
Gelatin	2.50g
Dextrose	0.25g
Sucrose	0.25g
Deionized Water	250mL

Supplementary Table 3. Universal 16S rRNA Gene Primers

Primer Name	Sequence (5' to 3')
Fwd-P1	*CCATCTCATCCCTGCGTGTCTCCGACTCAG*BBBBBBAGAGTTYGATYMTGGCTYAG
Fwd-P2	*CCATCTCATCCCTGCGTGTCTCCGACTCAG*BBBBBBAGARTTTGATCYTGGTTCAG
Rev1B	*CCTATCCCCTGTGTGCCTTGGCAGTCTCAG*ATTACCGCGGCTGCTGG

Titanium sequencing adaptors are italicized. The unique variable 6-9 base barcode is underlined, followed by the 5' end of the primer. Forward primers were combined in a 4:1 ratio (Fwd-P1:Fwd-P2).[27]

Supplementary Table 4. Genome Characteristics of *Megasphaera* phylotype 1 and *Megasphaera* phylotype 2 Isolates

	M1-70 (MP1)	28L (MP1)	UPII 199-6 (MP1)	M2-4 (MP2)	M2-8 (MP2)	UPII 135-E (MP2)
**Isolation Location**	VCU	JCVI	JCVI	VCU	VCU	JCVI
**Predicted Genome Size (Mb)**	1.78	1.73	1.64	1.74	1.71	1.65
**GC Percentage**	46.33	46.05	46.37	38.94	39.09	38.88
**Number of Contigs**	129	34	45	311	328	49
**N50 length (bp)**	179993	156177	100595	102411	131070	64000
**Number of Contigs @ N50**	4	5	7	6	5	8
**Transcriptome Size (Mb)**	1.55	1.55	1.46	1.46	1.41	1.44
**Transcriptome/Genome Ratio**	0.872	0.899	0.888	0.839	0.830	0.875
**Number of Predicted Genes**	1647	1715	1457	1591	1508	1510

Supplementary Table 6. Conserved Signature Indel and Conserved Signature Protein Analysis of Vaginal *Megasphaera* Phylotypes

	Megasphaera Phylotype 1 (MP1) strain 28L	*Megasphaera* Phylotype 1 (MP1) strain UPII 199-6	*Megasphaera* Phylotype 1 (MP1) strain M1-70	*Megasphaera* Phylotype 2 (MP2) strain UPII 135-E	*Megasphaera* Phylotype 2 (MP2) strain M2-4	*Megasphaera* Phylotype 2 (MP2) strain M2-8
**Conserved Signature Indels**						
Specific to Class *Negativicutes*						
3-Isopropylmalate dehydratase, large subunit (1 aa deletion, position 30-71)	Present	Present	Present	Present	Present	Present
DNA-directed RNA polymerase, subunit sigma (1 aa insertion, position 47-73)	Present	Present	Present	Present	Present	Present
Specific to Family *Veillonellaceae*						
GTP disphosphokinase (1 aa deletion, position 441-476)	Present	Present	Present	Present	Present	Present
GTP disphosphokinase (1 aa deletion, position 362-403)	Present	Present	Present	Present	Present	Present
**Conserved Signature Proteins**						
Specific to Class *Negativicutes*						
SELR_02010	Present	Present	Present	Present	Present	Present
SELR_03110	Present	Present	Present	Present	Present	Present
SELR_03270	Present	Present	Present	Present	Present	Present
SELR_05060	Present	Present	Present	Present	Present	Present
SELR_08460	Present	Present	Present	Present	Present	Present
SELR_10260	Absent	Absent	Absent	Absent	Absent	Absent
SELR_10270	Absent	Absent	Absent	Absent	Absent	Absent
SELR_15360	Absent	Absent	Absent	Absent	Absent	Absent
SELR_06480	Present	Present	Present	Present	Present	Present
Specific to Family *Veillonellaceae*						
MELS_0132	Present	Present	Present	Present	Present	Present
MELS_0206	Present	Present	Present	Present	Present	Present
MELS_0844	Present	Present	Present	Present	Present	Present
MELS_2049	Present	Present	Present	Present	Present	Present

*Presence of Conserved Signature Proteins (CSPs) and genomic regions containing Conserved Signature Indels (CSIs) indicative the class Negativicutes and the family Veillonellaceae are shown. All CSIs for both the class and family were detected in MP1 and MP2 genomes. All CSPs indicative of the family Veillonellaceae were also identified. Absent CSPs (3/9 indicative of the class Negativicutes) are denoted in red.

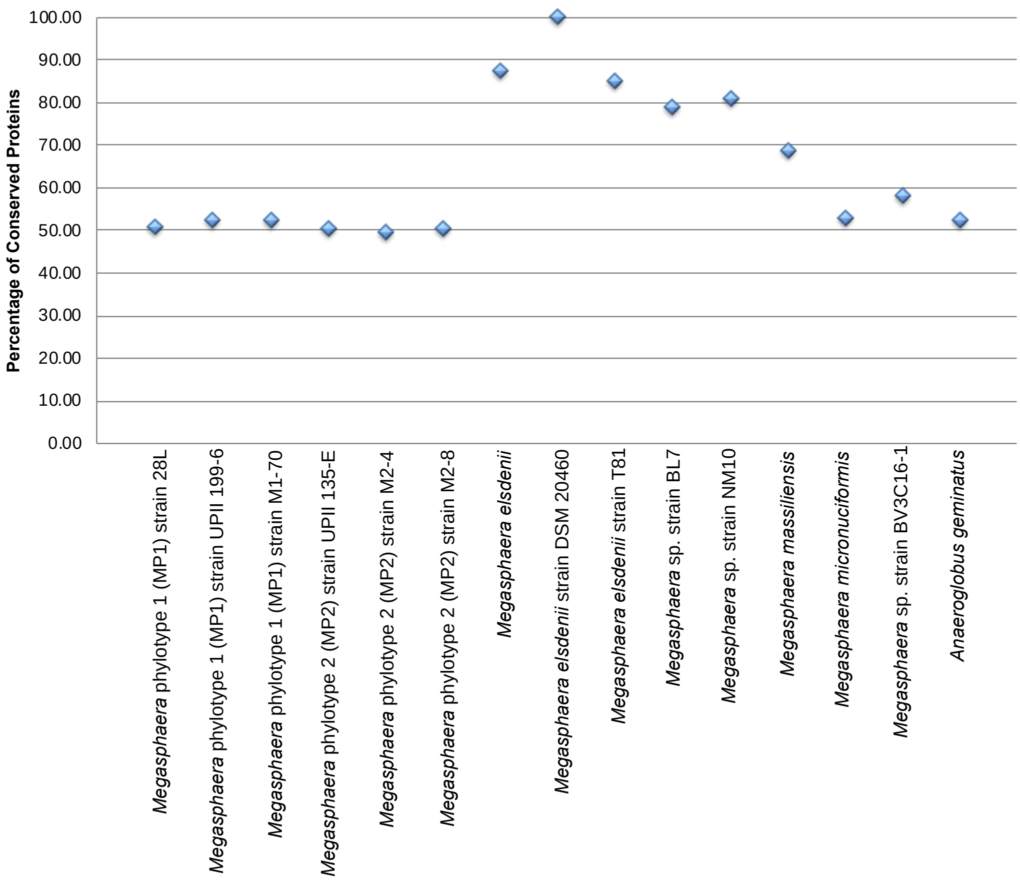

Supplementary Figure 1. Percentage of Conserved Proteins Analysis versus *Megasphaera* Type Strain. Pairwise Percentage of Conserved Proteins (POCP) values generated between 15 taxa and the *Megasphaera* type strain *Megasphaera elsdenii* strain DSM20460 are shown. POCP values below 50% are the suggested cutoff for delineation of a separate bacterial genus.

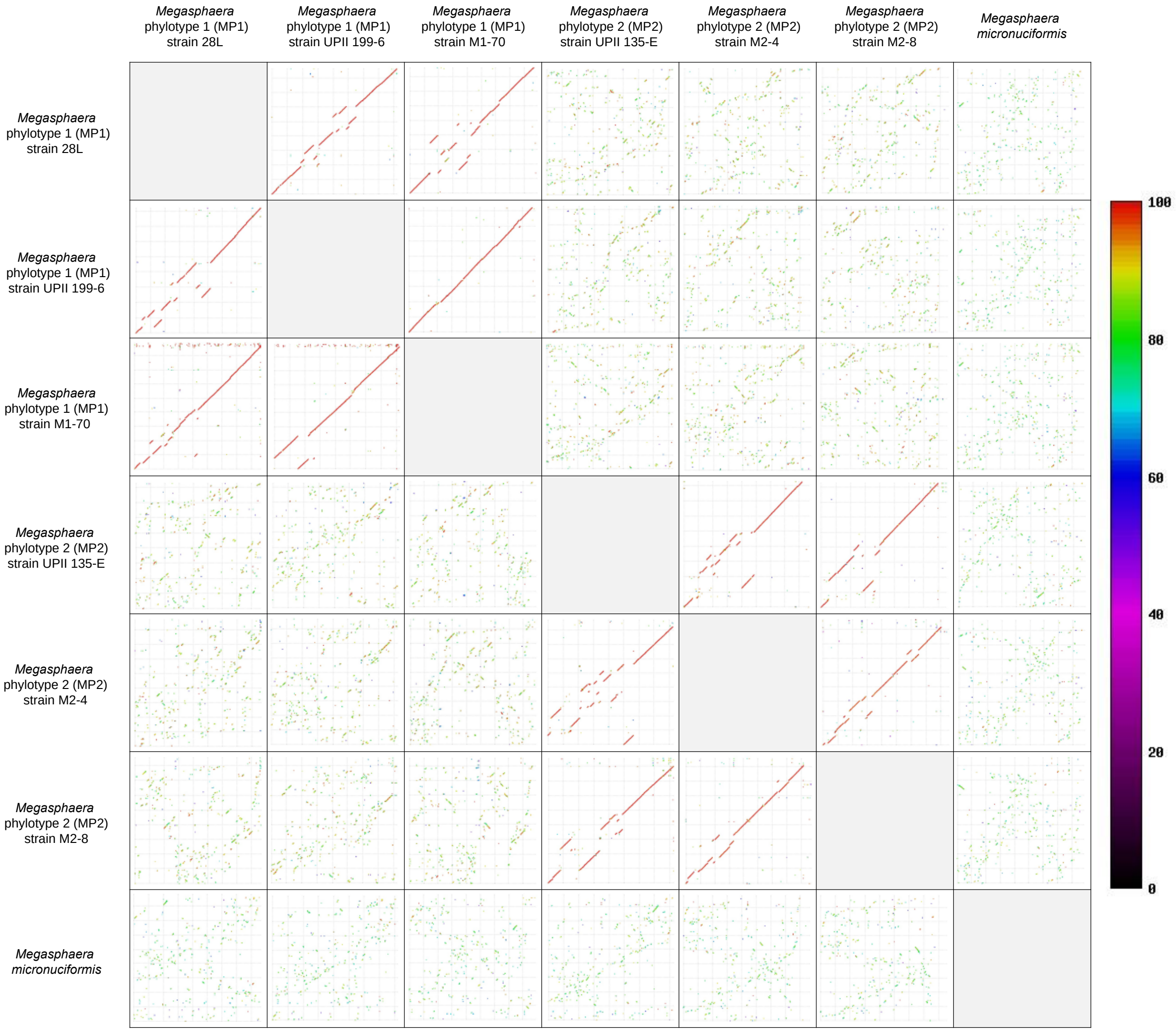

Supplementary Figure 2. Syntenic Comparison of Vaginal *Megasphaera* Phylotypes and the Oral Isolate *M. micronuciformis*. Full genomes for three MP1, three MP2 and one *Megasphaera micronuciformis* isolate were used for this analysis. Synteny plots demonstrate structural alignment of genomic content at the amino acid level. Color designates similarity at the amino acid level. Synteny is conserved within phylotype as evidenced clear alignment of genomes and protein identity is conserved as well. Between the two phylotypes and in comparsion of *M. micronuciformis*, massive genome rearrangement and loss of amino acid sequence conservation is observed.
